# Assessment of Artificial Intelligence-based Translation Tools for Emergency Department Discharge Instructions

**DOI:** 10.5811/westjem.48825

**Published:** 2026-04-08

**Authors:** Estee Wu, Cassandra Mackey, Simi Jandu, Jennifer L. Carey

**Affiliations:** * Upstate Medical University Norton College of Medicine, Syracuse, New York; † University of Massachusetts Chan Medical School, Department of Emergency Medicine, Worcester, Massachusetts

## Abstract

**Introduction:**

Emergency departments (ED) in the United States serve as a safety net for millions, including those with limited English proficiency (LEP). Eight percent of individuals living in the United States have LEP, placing them at risk for language barriers that can adversely affect the quality and safety of their care. Many hospitals lack language-concordant care, especially at the time of discharge. Miscommunication at discharge can lead to adverse health outcomes, including medication errors, poor compliance, and unnecessary return visits to the ED. Our objectives in this study were to evaluate the quality and safety of artificial intelligence (AI)-generated translations of physician-written, patient-specific ED discharge instructions and to assess performance across varying levels of instruction complexity.

**Methods:**

Emergency physicians wrote free-form discharge instructions, representing patient-specific guidance, which are typically provided at the time of ED discharge. Four topics were selected: abdominal pain; chest pain; wrist fracture; and vaginal bleeding in pregnancy. These instructions were intentionally developed to vary in linguistic complexity and were assessed using the Flesch Reading Ease and Flesch-Kincaid Grade Level scales. Instructions were translated into Albanian, Brazilian Portuguese, and Vietnamese using the AI-based translation tools ChatGPT-4, Microsoft Copilot, and Google Translate. Translations were evaluated for semantic and syntactic accuracy. Criteria included adequacy, fluency, meaning, and severity on a 5-point scale (1 = lowest accuracy, 5 = highest accuracy). Preference and formality were rated on a 3-point scale (1 = lowest, 3 = highest). The primary outcome was the quality and safety of AI-generated translations of patient-specific discharge instructions. Secondary outcomes included the ability to handle varying instruction complexity. Professional medical translators primarily responsible for the written translation of medical text evaluated and scored the translations for accuracy and quality metrics.

**Results:**

Overall adequacy, fluency, meaning, and severity scores were similar across models. ChatGPT-4 (3.79), Microsoft Copilot (3.60), and Google Translate (3.50), showed no statistically significant differences. Albanian translation was an exception, with ChatGPT-4 scoring significantly higher (3.75) than Google Translate (3.19) (P < .001). There were no other significant differences observed for Brazilian Portuguese or Vietnamese. ChatGPT-4 was also found to be the highest rated for Albanian and Brazilian Portuguese. Both Microsoft Copilot and Google Translate produced a total of five potentially harmful translation errors, whereas none were identified for ChatGPT-4.

**Conclusion:**

Miscommunication during discharge can lead to negative patient outcomes. This study evaluated ChatGPT-4, Microsoft Copilot, and Google Translate in translating ED instructions into Albanian, Brazilian Portuguese, and Vietnamese. ChatGPT-4 performed best overall and produced no harmful translations, and significantly outperformed Google Translate in Albanian. While AI-based translation tools show promise, human oversight remains necessary to mitigate risks from translation inaccuracies.

## INTRODUCTION

Over 25 million Americans have limited ability to read, speak, write, or understand English.^1^ Effective communication is crucial in healthcare, and barriers from limited English proficiency (LEP) lead to challenges in accessing equitable and quality healthcare. Entities receiving federal financial assistance are required by law to provide free language interpreting services for patients who have LEP.^2^,^3^ Written discharge instructions provide critical information regarding diagnoses, follow-up care, medication use, and when to seek further medical attention. Miscommunication at discharge can lead to adverse health outcomes, including medication errors, poor compliance, and unnecessary return visits to the emergency department (ED).^4^–^8^ Although providing discharge instructions in a patient’s preferred language is critical for ensuring comprehension, adherence, and safety, a recent study found that only 8% of patients with a non-English language preference received discharge instructions in their preferred language.^9^

Recent advancements in artificial intelligence (AI) and machine translation technologies offer the potential for more accurate and contextually appropriate translations. Translation systems driven by AI may be used for language translation, but prior studies have demonstrated variable success in medical translations.^10^–^12^ Advisories caution against the use of automated translation programs as they may provide erroneous or nonsensical translations. This can lead to misunderstandings and potentially compromise patient safety. Section 1557 of the Affordable Care Act (ACA) and the Code of Federal Regulations (CFR) 45 92.201 both state that translations must be reviewed by a qualified human translator to ensure accuracy.^2^,^3^

Our objectives in this study were to evaluate the quality and safety of AI-generated translations of physician-written, patient-specific ED discharge instructions and to assess performance across varying levels of instruction complexity. We examined emergency medicine (EM) discharge instructions translated into Albanian, Brazilian Portuguese (hereafter referred to as “Portuguese”), and Vietnamese using Chat Generative Pre-trained Transformer-4 (ChatGPT-4) (OpenAI, San Francisco, CA), Microsoft Copilot (Microsoft Corporation, Redmond, WA), and Google Translate (Google, LLC, Mountain View, CA). We used these three languages as representatives from different language families or subgroups reflective of the patient demographic in our region: Indo-European and Austroasiatic. To our knowledge, no prior studies have compared these AI-based translation tools across the three target languages based on the domains examined in this study.

## METHODS

### Study Setting

This study was carried out at an urban, academic medical center. In the ED at the time of discharge, patients receive instructions that include free-form, patient-specific information written by the physician. For this study, free-form discharge instructions were written and then translated by AI-based translation tools into three different languages reflective of the patient demographic in our region: Albanian; Portuguese; and Vietnamese. These written instructions were reviewed and assessed by the institution’s medical translators. This study was deemed not human subject research by our institutional review board.

**Population Health Research Capsule**

What do we already know about this issue?

*Limited comprehension of written discharge instructions due to language barriers is associated with medication errors, poor adherence, and unnecessary return visits*.

What was the research question?

*Do artificial intelligence (AI) tools safely and accurately translate patient-specific emergency department (ED) discharge instructions across languages?*

What was the major finding of the study?

*Overall scores were similar; ChatGPT-4 had zero harmful errors and outperformed Google Translate in Albanian (P < .001)*.

How does this improve population health?

*Although human oversight remains necessary, AI translation holds potential to improve ED discharge instructions for patients with limited English proficiency*.

### Discharge Instruction Development

Four sets of representative discharge instructions were written in a free-form style by a practicing emergency physician, followed by review and refinement by the remaining study investigators. These provided patient-specific information on diagnosis, treatment plans, and follow-up care at the time of ED discharge. The instructions were based on common clinical diagnoses treated in our ED: abdominal pain; chest pain; wrist fracture; and vaginal bleeding in pregnancy. Each was adapted from real instructions given to patients being discharged from our ED. The discharge instructions ranged from 65–109 words, with each set of instructions containing 5–7 sentences. Each discharge instruction contained the chief complaint, explanation of the chief complaint, workup results, management, and follow-up instructions. An example set of discharge instructions and corresponding translation by AI-based translation tools is reported in Appendix Table 1.

In writing the instructions, the aim was to capture variability in linguistic complexity. Instructions were scored based on readability measured by the Flesch Reading Ease (FRE) and Flesch-Kincaid Grade (FKG) level scales.^13^ The FRE and FKG scores for the instructions are reported in Appendix Table 2. The FRE scores ranged from 58.2 (fairly difficult to read), to 69.5 (easily understood by people 13–15 years of age). The FKG scores ranged from 6^th^–9th grade reading levels. We limited the analysis to four sets of discharge instructions to ensure feasibility of detailed expert review while preserving diversity in clinical content and linguistic complexity.

### Discharge Instruction Translations and Review

Written instructions were copied into ChatGPT-4, Microsoft Copilot, and Google Translate. The AI-based translation tools were accessed using the developers’ default public chat version available in September 2024. Each AI-based translation tools was prompted to translate the discharge instruction into Albanian, Portuguese, and Vietnamese. Instructions were then reviewed by professional medical translators employed by our institution in accordance with recommendations by the ACA and CFR.^2^,^3^ Medical translators are expertly trained in the written translation of medical content and documents and demonstrate a high level of language proficiency to accurately convert written documents and prevent errors. Medical translators are distinct from medical interpreters; interpreters facilitate spoken communication and convey meaning accurately and efficiently in real time.

Each translator received instructions in English and in their language of expertise. Translators were blinded to the AI-based translation platform used for translations and to the study objectives. A translator for each language reviewed all discharge instructions produced by each AI-based translation platform.

The primary outcome of the study was the quality and safety of AI-generated translations of patient-specific instructions written by the physician at the time of ED discharge. Secondary outcomes included the ability to handle varying instruction complexity. Scoring rubrics, adapted from Chen et al and Kanna et al, are shown in Appendix Table 3.^14^,^15^ Translations were assessed and scored by the institution’s translators for semantic accuracy (reflecting clarity of meaning) and syntactic accuracy (reflecting grammar, sentence structure, and formatting). Adequacy (estimated amount of information conveyed as compared to the untranslated text); fluency (grammar and understandability of a given text); meaning (the degree to which the translated text preserved content); severity (the degree of potential harm that could be caused to a patient receiving a given translated text); and preference (a measure to capture the translators’ subjective input regarding which translated version they preferred) were rated on a 5-point scale (1 = lowest accuracy, 5 = highest accuracy). Preference and formality were rated on a 3-point scale (1 = lowest, 3 = highest). Specific characteristics of the translation, rated on a scale of 0 (causing danger to the patient) to 2 (accurate, no safety concern) were based on accuracy of translation leading to safety concerns regarding the use of proper nouns, abbreviations, unconventional use of normal words, explanation of diagnosis/results, follow-up instructions, medication instructions, and return precautions.

### Data Analysis

We analyzed data using Prism software v10.4.2 (GraphPad, Inc, San Diego, CA). Descriptive statistics were calculated for all translation quality ratings, and we used one-way analysis of variance to assess differences among the three AI-based translation tools. A *P* < .05 was considered statistically significant.

## RESULTS

### Translation Evaluations

**Figure 1:**
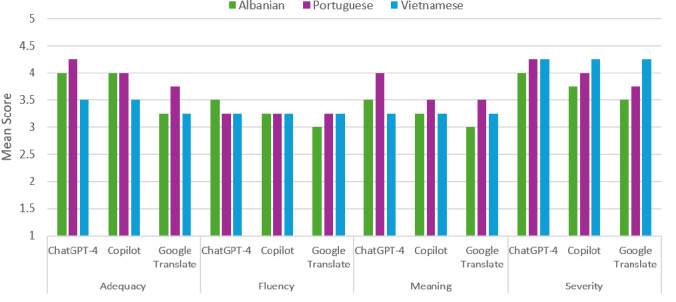
Mean score for adequacy, fluency, meaning and severity from artificial intelligence-based translation tools (ChatGPT-4, Copilot, and Google Translate) in a study where professional translators rated discharge instructions in Albanian, Brazilian Portuguese, and Vietnamese.

Four distinct discharge instructions were translated into three different languages. Overall mean scores across all languages and levels of reading difficulty for adequacy, fluency, meaning, and severity were as follows: ChatGPT-4 (3.79); Microsoft Copilot (3.60); and Google Translate (3.50) (*P* = .05). ChatGPT-4 (3.75) performed significantly better in Albanian compared to Google Translate (3.19) (*P* = .01); no other significant differences were seen across categories or when comparing FRE and FKG. Figure 1 shows breakdown by category.

Translators described text as harmful when acronyms were mistranslated or left unexplained, or when there was risk guiding patients to misunderstand key symptoms or delaying next steps in care. Translators noted the use of unfamiliar medical jargon, culturally incongruent terms, or ambiguous acronyms to be potentially harmful to patients with LEP or limited health literacy. Both Microsoft Copilot and Google Translate produced a total of five potentially harmful translation errors, whereas none were identified for ChatGPT-4. Specific comments for Albanian and Portuguese regarding errors in translations and miscommunication are shown in Table 1. There were no specific comments from Vietnamese translations.

#### Albanian

Overall, ChatGPT-4 had higher quality ratings with a mean of 3.75 compared to Microsoft Copilot (3.56). and Google Translate (3.19), with a significant difference observed between ChatGPT-4 and Google Translate (*P* < .001). Table 2 shows the comparison of platforms for each language. ChatGPT-4 was the most preferred for all but one discharge instruction set, while Google Translate was the least preferred.

**Figure 2:**
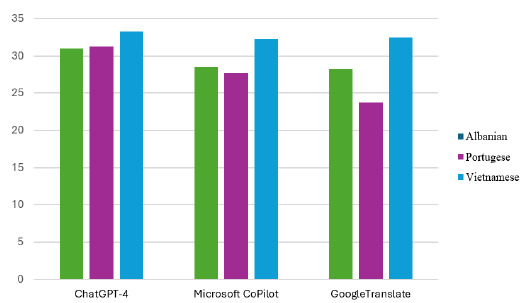
Total mean scores across artificial intelligence-based translation tools in a study comparing their accuracy in translating emergency department discharge instructions.

For specific characteristics, ChatGPT-4 scored the highest and Google Translate scored the lowest in explanation of diagnosis/results and follow-up instructions. Both Google Translate and Microsoft Copilot had two sets of instructions that posed potential harm to the patient in the categories “Explains diagnosis/results” and “Translation of abbreviations,” whereas ChatGPT-4 had none. Overall, ChatGPT-4 had the highest mean total score (31.25), followed by Microsoft Copilot (27.75) and Google Translate (23.75) (Figure 2).

#### Portuguese

There was no significant difference between the three AI-based translation tools. For ChatGPT-4, two discharge instructions were rated as “most preferred” while Google Translate had two discharge instructions rated as “least preferred.” Google Translate was rated the least formal for three of the four discharge instructions, with ChatGPT-4 being the most formal for two of the four discharge sets. All AI-based translation tools were rated similarly for proper use of nouns, follow-up, medications instructions, and return precautions. Both Google Translate and Microsoft Copilot had one set of instructions finding potential harm to the patient in the categories “Explains diagnosis/results” and “Translation of abbreviations,” whereas ChatGPT-4 had none. Overall, ChatGPT-4 was rated the highest overall at a mean total score (31), followed by Microsoft Copilot (28.5) and Google Translate (28.25).

#### Vietnamese

There was no significant difference between the three AI-based translation tools. Across all instructions, none of the AI-based translation tools stood out for preference and formality; and for specific characteristics of the discharge instructions, the mean scores across all three AI-based translation tools were equal. There were no Vietnamese translations rated as potentially harmful to patients. Overall, ChatGPT-4 had the highest mean total score (33.25) followed by Google Translate (32.5) and Microsoft Copilot (32.25).

#### Albanian

Compared with Vietnamese and Portuguese, Albanian AI-generated translations demonstrated significant differences in tool performance, with ChatGPT-4 scoring significantly higher than Google Translate (p<.001). In contrast, no significant differences were observed among translation tools for Portuguese and Vietnamese, suggesting greater variability in platform performance for Albanian translations. Notably, ChatGPT-4 produced no translations posing potential harm in Albanian, whereas Google Translate and Microsoft Copilot had two sets of instructions that posed potential harm. For Portuguese, both Microsoft Copilot and Google Translate produced one instruction set containing potential harmful content, whereas none were identified for Vietnamese translations.

## DISCUSSION

Instructions at the time of discharge are critical for patient care and safety. Miscommunication due to language barriers, improper instruction delivery, or lack of comprehension, can lead to adverse health outcomes such as unplanned revisits, medication errors, and increased readmission rates.^4^–^7^ Improving discharge communication is crucial to enhance patient safety and overall health management. 5–8

We evaluated three widely used AI-based translation tools—ChatGPT-4, Microsoft Copilot, and Google Translate—in translating ED discharge instructions into Albanian, Portuguese, and Vietnamese. In this study, ChatGPT-4 demonstrated the strongest overall performance compared with the others but did not reach statistical significance, with the exception of ChatGPT-4 compared to Google Translate in Albanian. ChatGPT-4 produced no harmful translations, whereas the other two models each generated potentially harmful output in two categories. ChatGPT-4 also achieved the highest scores for critical instruction components such as diagnosis explanation and follow-up recommendations, suggesting greater contextual awareness in more complex and nuanced translation tasks. Comments from translators highlight consistent issues across all platforms. All three tools were reported to contain poor syntax, incorrect or unclear translations of medical acronyms and terminology, and contextually inappropriate word choices, which may have led to confusion for patients. The severity scores for Vietnamese suggested that critical aspects of patient comprehension and safety were largely preserved. Despite this, the models scored modestly on preference and formality, indicating that the tone and stylistic appropriateness of translations may not meet patient or clinician expectations. The greatest variability in model performance was observed in Albanian translations. Factors such as healthcare-specific training data is less abundant and available to AI-based translation tools.

We used representative ED discharge instructions that closely mirror real-world clinical practice and incorporated different readability levels based on the FRE and FKG scales. We compared three widely used AI-based translation tools, relying on professional medical translators responsible for producing accurate translations that are understandable to patients. Our study, like others, found variability among AI-based translation tools and languages. A study of standardized pediatric discharge instructions found that Google Translate and ChatGPT performed similarly to professional translators for Portuguese, with low rates of clinically significant errors.^11^ A study translating postoperative discharge instructions for circumcisions and patient information for undescended testicles found that ChatGPT has an unacceptably high rate of translation error in Vietnamese.^16^

Another study assessing 20 commonly used ED discharge instruction phrases found that Vietnamese scored 77.5% for accuracy by volunteer native speakers.^12^ Studies examining ED discharge instructions indicate that Google Translate’s accuracy varies by language. One study reported it accurately translated 92% of Spanish and 81% of Chinese sentences, while another study showed Spanish translation accuracy was highest (94%) followed by Tagalog (90%), Korean (82.5%), Chinese (81.7%), Farsi (67.5%), and Armenian (55%).^6^,^10^ Other studies have looked at AI-based translation tool for translation of standardized discharge instructions and have similarly found variation by languages, with worse performance in Haitian Creole, Russian, and Vietnamese.^11^,^16^ To our knowledge, no prior studies have examined the use of AI-based translation tools for translating discharge instructions into Albanian.

Translation tools assisted by AI show promise when other forms of translation services have limited availability. However, at this time they must still be used with caution and under human supervision. Even infrequent errors, such as mistranslations of abbreviations, diagnostic terms, or medication instructions, can pose significant risks to patient outcomes. Advancements in AI technology offer the potential for improved translation accuracy and contextual relevance. It is essential to make physicians aware of the limitations of this technology and to continue to conduct ongoing evaluations across diverse clinical contexts and languages.

## LIMITATIONS

This study assessed four discharge instructions translated into three languages; therefore, it may not capture the full range of complexity across all languages or the broader spectrum of FRE and FKG scales. Languages with more complex grammar, longer word forms, or different writing systems, such as tonal languages or right-to-left scripts, may present unique challenges not captured here. Additionally, while readability was evaluated using FRE and FKG scales, these measures primarily focus on sentence length and word complexity but do not account for health literacy. Instructions were created and reviewed within a single institution, which may limit generalizability to other clinical settings or patient populations.

## CONCLUSIONS

This study evaluated ChatGPT-4, Microsoft Copilot, and Google Translate in translating four representative ED discharge instructions into three languages: Albanian, Portuguese, and Vietnamese. ChatGPT-4 performed best overall, conveying critical medical details without harmful errors, although all tools struggled with syntax and medical terminology. While AI-based translation tools show promise, they still require human oversight due to potential translation errors. Due to the limited sample size, further studies are needed to determine which AI-based translation tools perform best.

## Supplementary Information



## Figures and Tables

**Table 1:** Translators’ comments on linguistic discrepancies in artificial intelligence translations of Brazilian Portuguese and Albanian discharge instructions.

Language	Chief complaint	ChatGPT-4	Microsoft Copilot	Google Translate
Portuguese	Abdominal pain	Added an explanation to the condition’s acronym Didn’t translate “PCP” in a way that is widely understood by Brazilians	Kept the acronym in English (Brazilians don’t use acronyms for medical conditions) Didn’t translate “PCP” in a way that is widely understood by Brazilians	Translated the English acronym to the Portuguese one, which patients don’t recognize or understand Kept “PCP” in English
	Chest pain	Poor syntax One of the tests was not translated into Portuguese The medication name should remain in English for easy identification at the pharmacy	Poor syntax Tests were not translated into Portuguese The translation of “emergency department” is not commonly used in Brazil The medication name should remain in English for easy identification at the pharmacy.	Poor syntax Tests were not translated into Portuguese The medication name should remain in English for easy identification at the pharmacy.
	Wrist pain	Poor syntax The medication name should remain in English for easy identification at the pharmacy	Poor syntax The medication name should remain in English for easy identification at the pharmacy The translation of “emergency department” is not commonly used in Brazil.	Poor syntax The medication name should remain in English for easy identification at the pharmacy.
	Vaginal bleeding	No issues that would cause major patient misunderstanding or confusion	A literal translation was used, which does not clearly convey the intended meaning The translation of “emergency department” is not commonly used in Brazil	The word “date” has multiple meanings, and it was not translated correctly based on the context, which is concerning as it may lead to confusion
Albanian	Abdominal pain	Keeps acronyms, added an explanation to the condition’s acronym Didn’t translate “PCP” in a way that clarifies which doctor to call	Kept the acronyms of CT and diagnosis without translating them, which wouldn’t be recognized and/or understood Acronyms for medical conditions are not used in Albanian. Didn’t translate “PCP” in a way that is clear	Kept the acronyms of CT and diagnosis without translating them, which wouldn’t be recognized and/or understood Acronyms for medical conditions are not used in Albanian language, Kept “PCP” in English
	Chest pain	One of the tests’ abbreviations is not translated into Albanian. The name of the test is translated in an unconventional way and unclear. Translation of the diagnosis is not accurate. The medication name should remain in English to allow for identification at the pharmacy. Translation of the word “reassuring” is not correct. Poor syntax	Tests’ abbreviations are not translated into Albanian, Poor translation Translation of the diagnosis is not accurate. The medication name should remain in English to allow for identification at the pharmacy. Translation of the word “reassuring” is not correct. Poor syntax	Tests’ abbreviations are not translated into Albanian, Translation of the diagnosis is not accurate. The medication name should remain in English to allow for identification at the pharmacy. Translation of the word “reassuring” is not correct. Poor syntax
	Wrist pain	The name of the test is translated in an unconventional way and unclear. Translation of the diagnosis is not accurate. Poor translation of medical terminology The medication name should remain in English to allow for identification at the pharmacy Poor syntax	The name of the test is translated in an unconventional way and unclear. Translation of the diagnosis is not accurate. Incorrect medical terminology The medication name should remain in English to allow for identification at the pharmacy. Poor syntax	The name of test is translated in an unconventional way and is incorrect and iunclear. Translation of the diagnosis is not accurate. Incorrect medical terminology Poor syntax
	Vaginal bleeding	The name of the test is translated in an unconventional way and is unclear. Omits the word fetal when translating “fetal heart rate”		The name of test is translated in an unconventional way and is incorrect and unclear The word “dates” is translated as appointments which is incorrect for the context and leads to confusion for the patient.

*AI*, artificial intelligence; *PCP*, primary care physician; *CT*, computed tomography.

**Table 2:** Mean quality rating scores of artificial intelligence-based translation tools based on language.

Language	AI-based translation tool			*P*-value

	ChatGPT4	Microsoft Copilot	Google Translate	
Albanian	3.75	3.56	3.19	.02
Portuguese	3.94	3.69	3.63	.31
Vietnamese	3.69	3.63	3.69	.95

*AI*, artificial intelligence.
