# Supplementary material for: Assessment of Artificial Intelligence-based Translation Tools for Emergency Department Discharge Instructions
Source: West J Emerg Med. 2026 Apr 8;27(3):651–8. doi: 10.5811/westjem.48825 (PMC13246204; doi:10.5811/westjem.48825)
Supplement: Supplementary file 1 [file wjem-27-651-s001.docx]

**Appendix**

**Table 1.** Sample Discharge Instruction and Translation to Albanian by AI-based translation tools

| English | You were seen here today for your chest pain. Your labs, EKG, and CXR were reassuring but you have a condition called stable angina. This is chest pain that occurs with exercise or emotional stress. A prescription for nitroglycerin has been sent to your pharmacy, please take as directed on the bottle. If you have worsening chest pain, shortness of breath at rest, please return to the Emergency Department. |
| --- | --- |
| Chat GPT-4 | Ju u kontrolluat sot për dhimbje në gjoks. Analizat e gjakut, EKG-ja dhe rrezet X të kraharorit ishin qetësuese, por ju keni një gjendje të quajtur anginë stabile. Kjo është një dhimbje gjoksi që ndodh gjatë ushtrimeve fizike ose stresit emocional. Një recetë për nitroglicerinë është dërguar në farmacinë tuaj, ju lutem merreni sipas udhëzimeve në shishe. Nëse keni përkeqësim të dhimbjes në gjoks, ose vështirësi në frymëmarrje në qetësi, ju lutem kthehuni në repartin e Urgjencës. |
| Microsoft Copilot | Ju u patë sot për dhimbje gjoksi. Analizat tuaja, EKG dhe CXR ishin qetësuese, por keni një gjendje të quajtur angina stabile. Kjo është dhimbje gjoksi që ndodh me ushtrime ose stres emocional. Një recetë për nitroglicerinë është dërguar në farmacinë tuaj, ju lutem merrni sipas udhëzimeve në shishe. Nëse keni dhimbje gjoksi të përkeqësuar, vështirësi në frymëmarrje në pushim, ju lutem kthehuni në Departamentin e Urgjencës. |
| Google Translate | Ju u patë këtu sot për dhimbje gjoksi. Laboratorët tuaj, EKG dhe CXR ishin qetësuese, por ju keni një gjendje të quajtur angina e qëndrueshme. Kjo është dhimbje gjoksi që shfaqet me stërvitje ose stres emocional. Një recetë për nitroglicerinë është dërguar në farmacinë tuaj, ju lutemi merrni siç udhëzohet në shishe. Nëse keni dhimbje gjoksi të përkeqësuar, gulçim në pushim, ju lutemi kthehuni në Departamentin e Urgjencës. |

*EKG*, electrocardiogram; *CXR*, chest X-ray

**Table 2.** Flesch-Kincaid Readability and Flesch-Kincaid Grade Level scale for sample discharge instructions

|  | Flesch-Kincaid Readability  (0= easier to read, 100= more difficult) | Flesch-Kincaid Grade Level Scale |
| --- | --- | --- |
| Sample Instruction #1 Abdominal Pain | 69.5 | 6.2 |
| Sample Instruction #2 Chest Pain | 66.5 | 7.4 |
| Sample Instruction #3 Wrist Fracture | 64.5 | 8.1 |
| Sample Instruction #4 Vaginal Bleeding in Pregnancy | 58.2 | 9.3 |

**Table 3A.** General scoring rubric

| **Score** | **1** | **2** | **3** | **4** | **5** |
| --- | --- | --- | --- | --- | --- |
| **Adequacy** | 0% of information conveyed from original | 25% of information conveyed from original | 50% of information conveyed from original | 75% of information conveyed from original | 100% of information conveyed from original |
| **Fluency** | No fluency; no appreciable grammar, not understandable | Marginal fluency; several grammatical errors | Good fluency; several grammatical errors | Excellent fluency; few grammatical errors | Perfect fluency; no grammatical errors |
| **Meaning** | Totally different meaning from the original | Misleading information added or omitted compared with the original | Partially the same meaning as the original | Almost the same meaning as the original | Same meaning as the original |
| **Severity** | Dangerous to patient | Impairs care in some way | Delays necessary care | Unclear effect on patient care | No effect on patient care |

**Table 3B**. Scoring rubric: platform preference

| **Score** | **Preference** | **Formality** |
| --- | --- | --- |
| 1 | Least Preferred | Least Formal |
| 2 | Between | Between |
| 3 | Most Preferred | Most Formal |

**Table 3C.** Scoring rubric: additional characteristics

|  | 0 | 1 | 2 |
| --- | --- | --- | --- |
| Use of Proper Nouns | Causes danger to patient | Inaccurate information but maintains pertinent information | Accurately translated, no safety or patient care concerns |
| Explains diagnosis/results |  |  |  |
| Follow-up instructions |  |  |  |
| Medication Instructions |  |  |  |
| Return Precautions |  |  |  |
| Unconventional Use of Normal Words |  |  |  |
| Translation of Abbreviations |  |  |  |
